# I. Tenotomy. II. Resection of Necrotic Parts of the Soft Tissues of the Frog for Nail-wound1Read before the semi-annual meeting of the Pennsylvania State Veterinary Medica Association, Franklin, September, 1897.

**Published:** 1897-10

**Authors:** Otto G. Noack

**Affiliations:** Reading, Pa.


### I. TENOTOMY. II. RESECTION OF NECROTIC PARTS OF THE SOFT TISSUES OF THE FROG FOR NAIL-WOUND.[1]

BY OTTO G. NOACK, V.S.,
READING, PA.

CASE I.—On December 3, 1896, I was called to see a bay horse, twelve years old, 16 hands high. The owner reported to me that

[1] Read before the semi-annual meeting of the Pennsylvania State Veterinary Medica Association, Franklin, September, 1897.

the horse had to pull a very heavy load about six months ago, and had limped ever since. By and by the leg shortened, and the horse began to walk on the toe, then got worse, being unable to walk at all. Upon examination I found excessive volar flexion of the left front hoof in the coffin-joint, caused by contraction of the flexor perforans. In the region between the first and second third of the metacarpus there could be noticed a thickening in the form of a hard and sharply-defined swelling. My diagnosis was chronic tendinitis. I informed the owner that the prognosis was not very favorable, and the only treatment to be applied was tenotomy. As the horse in that condition was worthless, the owner consented.

As the general practitioner has not much time to lose, and the operations have to be made with little assistance, it is an impossibility to go according to rules and lines, and one has to be guided by circumstances. I at first disinfected the leg as well as was possible under the circumstances, injected right above the thickening 5 grammes of a 10 per cent. solution of cocaine, and, putting the twitch to the horse's upper lip, I made an incision through the skin with a pointed bistoury right at the swelling on the outside, and after this I inserted a blunt tenotome, with its surface lying close to the tendon, till I felt it on the inside, and turned the knife against the tendon, and, cutting it slowly, the hoof came down suddenly with a loud noise before the tendon was completely cut through, and the horse could stand on his foot. Profuse bleeding showed that bloodvessels had permeated the thickening already; after cleansing with a solution of creolin I applied an aseptic bandage. Two days afterward I opened the bandage and found that suppuration had set in, perhaps by hair or other foreign body that had gained access during the operation. The wound was cleaned every other day with creolin. After the third day of operation the horse got daily exercise for about ten minutes for three weeks. In this time the wound had healed, and the horse, by walking on the old shoes, had a very bad plantar flexion. To avoid this I gave the horse a shoe without calking, but thickened heels, the toe cut very short, with rolling motion, and by this I overcame the plantar flexion. The horse was put to work three weeks later, and is working ever since.

CASE II.—In the middle of January, 1897, I had to attend a horse which had tread on a nail some time before. The wound did not appear to heal, and occasioned great pain and caused the horse to walk very lame. I cut away some horn around the wound and recommended soaking the horse's foot in a bath of creolin. In four weeks I was called again, with the remark that the wound had healed, but the horse still remained lame. I recommended blistering around the coronary band, which was done, but without success. I then told the owner the only thing to be done was to operate, as there must be a lesion of the bone, with suppuration of necrotic parts of the tissues where the nail went through, but it was doubtful if the operation would be of avail. After two months' consideration I was instructed to operate upon the horse, as in this condition he was worthless.

On June 6th I performed the operation, casting the horse and paring the inner side of the frog off to the plantar cushion, and soon found the sore left by the nail, showing a black spot, reaching in the plantar cushion nearly three-fourths of an inch, of the same dark color, being necrosed. I removed the diseased parts and applied an aseptic bandage, and during four weeks I only changed the dressing four or five times, the wound healing without any suppuration whatever. The horse was put to work without showing any lameness.

---

The Board of Aldermen of New York City has adopted new rules for bicyclists and drivers on highways. The rules give to those going north and south the right of way over those going east and west, and make it a misdemeanor for a citizen not to yield the right of way to an ambulance, police wagon, fire-engine, or doctor's wagon, provided the doctor has a police permit. Riders of bicycles and drivers on highways must keep to the left when overtaking another going in the same direction, and above Thirty-ninth street they cannot turn unless fifteen feet above the vehicle behind. Drivers must raise their whips or give some kind of a signal when about to turn a corner or to stop. Bicyclists must carry a bell not more than four inches in diameter, so that they may not be mistaken for a fire-engine, ambulance, or a doctor. Eight miles an hour must be the limit of speed, and no more than two may ride abreast. Coasting is forbidden below One-hundred-and-twenty-fifth street. Bicycle-riders must carry lamps as at present. Drivers of vehicles must be more than sixteen years old; heavy wagons must not go more than five miles an hour on the streets, and vehicles other than bicycles are forbidden to turn corners faster than three miles an hour. Riding on the sidewalk is prohibited, but wheelmen may trundle their machines in single file. Bicyclists must not carry children less than five years old on their wheels. The penalty for violation of any of the rules is ten dollars.

